# The gut microbiome and Alopecia areata: Implications for early diagnostic biomarkers and novel therapies

**DOI:** 10.3389/fnut.2022.979876

**Published:** 2022-09-15

**Authors:** Yongbo Kang, Yue Cai, Yanqin Zhao, Ying Yang

**Affiliations:** ^1^Department of Endocrinology, Affiliated Hospital of Yunnan University, Kunming, China; ^2^Department of Microbiology and Immunology, School of Basic Medical Sciences, Shanxi Medical University, Taiyuan, China

**Keywords:** gut microbiota, Alopecia areata, autoimmune disease, biomarker, fecal microbiota transplantation

## Abstract

Alopecia areata (AA) accounts for the autoimmune disorder mediated by T cells, whose prognostic outcome cannot be predicted and curative treatment is unavailable at present. The AA pathogenic mechanism remains largely unclear, even though follicular attack has been suggested to result from that attack of immune privilege-losing hair follicles driven by immunity. Recently, gut microbiota is suggested to have an important effect on immunoregulation under autoimmune situations like AA. Fecal microbial transplantation (FMT) may be used to treat AA. Nonetheless, related research remains at the initial stage. To promote the rapid progress of relevant research, the present work aimed to shed more lights on gut microbiota's effect on AA, early diagnostic biomarker and FMT therapeutics.

## Introduction

Alopecia areata (AA) represents one of the skin diseases with the population incidence of 0.1–0.2%, and it will result in hair loss in most patients resistant to treatment. AA has adverse effects on self-esteem and body image that are critical for socialization, as a result, it will result in inefficient social functioning as well as reduced life quality (1, 2). What's more, some recent studies have demonstrated that AA can be associated with many types of disease, such as major depressive disorder, vitiligo, thyroiditis, atopic diseases, systemic lupus erythematosus (SLE), rheumatoid arthritis, psoriasis, stroke, cardiovascular disease and so on (3–5). Therefore, more attention needs to be paid to AA. At present, AA's pathogenic mechanism and etiology remain unclear. As revealed by experimental and clinical studies, AA is due to hair follicle attack by the autoimmunity, thus inducing hair follicular inflammation (6, 7). The autoimmunity is the defect in immune tolerance, resulting in the production and proliferation of autoreactive T-cells and antibodies. But factors related to autoimmunity within AA are unclear (8).

Gut microbiota has an important effect on human disorders and health. In particular, more and more evidence has demonstrated that its critical effect on autoimmune disorders like psoriasis, inflammatory bowel disease (IBD), and SLE (9–12). As one of the autoimmune disorders, AA is possibly related to gut microbial dysbiosis.

## Gut microbiota and AA and its potential for early diagnosis

So far, just several articles analyze the relation of gut microbiota with AA (Table 1). Rangu et al. (13) found that difference in β-diversity showed statistical significance in AA patients compared with the controls. Meanwhile, *Ruminococcus bicirculans* had reduced relative abundance among AA cases compared with normal subjects. According to a study analyzing the abundances of gene orthologs, there were 20 differential orthologs between 2 groups, including metal transport- and spore germination-related genes. Likewise, Lu et al. (14) found that AA patients had significantly different gut microbial communities compared with healthy controls. Furthermore, 3 AA-related OTU biomarkers, including OTU257 (*Megasphaera*), OTU1237 (*Achromobacter*), as well as OTU1784 (*Lachnospiraceae Incertae Sedis*), were identified. In keep with the observation, Moreno-Arrones et al. (15) discovered that *Erysipelotrichacea, Holdemania filiformis, Parabacteroides johnsonii, Parabacteroides distasonis, Lachnospiraceae, Bacteroides eggerthii*, and *Clostridiales vadin* BB60 group had higher abundances in patients affected with alopecia universalis (AU) compared with controls. Furthermore, a prediction model constructed according to *Clostridiales vadin* BB60 group and *Parabacteroides distasonis* bacterial quantities achieved an 80% accuracy in predicting the disease.

**Table 1:** Changes in oral microbiota composition associated with AA.

**Models**	**Disease**	**Implicated microbiota**	**References**
Human	AA	*Ruminococcus bicirculans*↓	(10)
Human	AA	*Achromobacter↑, Megasphaera↑,* *Lachnospiraceae Incertae Sedis↑*	(11)
Human	AU	*Holdemania filiformis*↑, *Erysipelotrichacea*↑, *Lachnospiraceae*↑, *Parabacteroides johnsonii*↑, *Clostridiales vadin* BB60 group↑, *Bacteroides eggerthii*↑, *Parabacteroides distasonis*↑	(12)

↑, increase; ↓, decrease.

All in all, gut microbiota has a critical effect on AA genesis and development, and their biomarkers may potentially be used as earlier diagnosis and therapeutic targets.

## Future prospect of fecal microbiota transplantation for AA treatment

Currently, AA preventive treatment is unavailable. Due to the great contribution of gut microbiota to AA occurrence, FMT has become the candidate target for preventive treatment. As far as we know, FMT is not specifically investigated for AA improvement. Just two case reports are conducted to depict hair growth among AA cases post-FMT. FMT has been recommended to treat infection with *Clostridium difficile*, which may also be used to treat non-alcohol fatty liver disease (NAFLD), IBD, irritable bowel syndrome, and obesity (16–19). Rebello et al. (20) found that hair regrew in 2 AU cases post-FMT to treat the relapsed infections with *C. difficile*. In keep with such observation, Xie et al. (19) found that a patient with AA who developed hair regrowth in the involved scalp, and a portion of gray hairs slowly turned into black after FMT for treatment of non-infectious diarrhea. Thus, FMT is the possible treatment for AA cases.

## Concluding remarks and perspectives

At present, some preliminary research suggests the tight relation between gut microbiota and AA. Therefore, gut microbiota may have immunomodulatory effects in AA, but more investigations are needed for elucidating the mechanism underlying AA. If the gut microbiota can be identified to be related with the immunity activity of AA, the differential gut microbiota may potentially be used to diagnose and judge the condition of AA in advance. Meanwhile, this may assist in development other treatments for AA cases who have restricted treatment options at present. FMT is the possible treatment for AA cases.

These studies are just beginning and there is a lot more research to be done. Firstly, more comprehensive research concerning gut microbiota-host interaction mechanism is warranted among diverse populations. Secondly, in order to diagnose AA earlier, longitudinal studies should be conducted on changes in gut microbiota during the development of AA. Thirdly, although FMT has been gradually standardized, the composition of gut microbiota required to treat different diseases is different. Therefore, the well-controlled clinical trials are needed to determine which types of gut microbiota can be used to treat AA. Furthermore, the optimal donors were screened by constructing biomarkers based on gut microbiota. Last but not least, in order to achieve individual precise treatment of AA, it is necessary to identify the specific species among gut microbiota which play either a protective or a pathogenic role in the progression of AA. All in all, further research in this area will open up an entirely new approach to the understanding and treatment of AA.

## Data availability statement

The original contributions presented in the study are included in the article/supplementary material, further inquiries can be directed to the corresponding author/s.

## Author contributions

YK, YC, and YZ collected the data and wrote most of the manuscript with help from YY. All authors have read and approved the final manuscript.

## Funding

This work was supported by the Natural Science Foundation of China (Grant No. 82160159), Science Research Start-up Fund for Doctor of Shanxi Medical University (Grant No. XD1807), Science Research Start-up Fund for Doctor of Shanxi Province (Grant No. SD1807), Scientific and Technological Innovation Programs of Higher Education Institutions in Shanxi (Grant No. 2019L0425), and Shanxi Province Science Foundation for Youths (Grant No. 201901D211314).

## Conflict of interest

The authors declare that the research was conducted in the absence of any commercial or financial relationships that could be construed as a potential conflict of interest.

## Publisher's note

All claims expressed in this article are solely those of the authors and do not necessarily represent those of their affiliated organizations, or those of the publisher, the editors and the reviewers. Any product that may be evaluated in this article, or claim that may be made by its manufacturer, is not guaranteed or endorsed by the publisher.
